# Tracheobronchopathia Osteochondroplastica: End Stage of Tracheo-Bronchial Amyloidosis?

**Published:** 2019-03

**Authors:** Haifa Zaibi, Rana Fessi, Besma Dhahri, Jihen Ben Amar, Hichem Aouina

**Affiliations:** Faculty of Medicine of Tunis, University of Tunis El Manar, Tunisia, Charles Nicole Pulmonology Department, Tunis, Tunisia

**Keywords:** Amyloidosis, Tracheobronchopathia osteochondroplastica

## Abstract

Tracheobronchopathia osteochondroplastica is a rare idiopathic disease of the trachea and the main bronchi, characterized by multiple submucosal osteocartilaginous nodules. Although the etiology of tracheobronchopathia osteochondroplastica remains unknown, several theories have been proposed. We report a case of a 47-year-old non-smoker woman with wheezing dyspnea over the past two years, which was treated as asthma without improvement. Investigations, including chest computed tomography scan, fiberoptic bronchoscopy, and endobronchial biopsy, indicated tracheobronchial amyloid light-chain (AL) amyloidosis. Thirteen years later, she was admitted for cough and wheezing. The bronchoscopic examination demonstrated nodular lesions distributed along the cartilaginous rings of the lower portion of the trachea and the main bronchi. Endobronchial biopsy confirmed tracheobronchopathia osteochondroplastica. We found tracheobronchopathia osteochondroplastica to be the end stage of amyloidosis.

## INTRODUCTION

Tracheobronchopathia osteochondroplastica (TO) is an uncommon benign disease, affecting the cartilaginous wall of large airways. Although the etiology of TO is unknown, review of the literature revealed a close interrelationship between TO and amyloidosis. Many researchers suggest that TO is the advanced stage of primary tracheobronchial amyloidosis.

Herein, we present a case of TO as the end stage of tracheobronchial amyloidosis.

## CASE SUMMARIES

**Figure 1.**
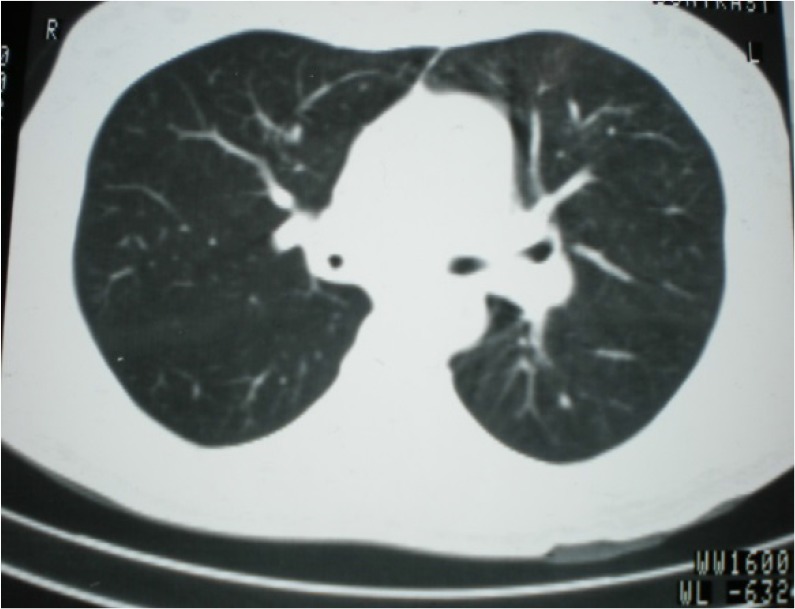
Chest tomography scan: Irregular thickening of wall of the right main bronchi

A 47-year-old non-smoker woman was admitted to our department with a two-year history of dyspnea and wheezing. The physical examination indicated diffuse sibilants, while the result of chest X-ray was normal. The pulmonary function test showed an obstructive pattern with forced vital capacity (FVC) at 78%, forced expiratory volume (FEV) at 43%, and FEV/FVC at 48%. Diagnosis of asthma was initially established. Inhaled β _2_ adrenergic agonist and corticosteroid were administered, without any improvements in the symptoms or pulmonary function test. Chest computed tomography (CT) scan revealed irregular thickening of the tracheal wall, with nodules protruding into the lumen of the right main bronchi and lower lobe bronchiectasis (Figure 1).

Fiberoptic bronchoscopy showed infiltration and nodular lesions, protruding into the lumen of the lower third portion of the trachea and the main bronchi. Diagnosis of tracheobronchial amyloid light-chain (AL) amyloidosis was confirmed by endobronchial biopsy. The specimen examination under polarized light with Congo red staining revealed green-yellowish amyloid deposits. Extensive investigations, including renal and hepatic ultrasonography, liver function test, creatinine clearance rate, serum and urine protein electrophoreses, and electro- and echocardiography, were normal, leading to the exclusion of systemic amyloidosis.

**Figure 2.**
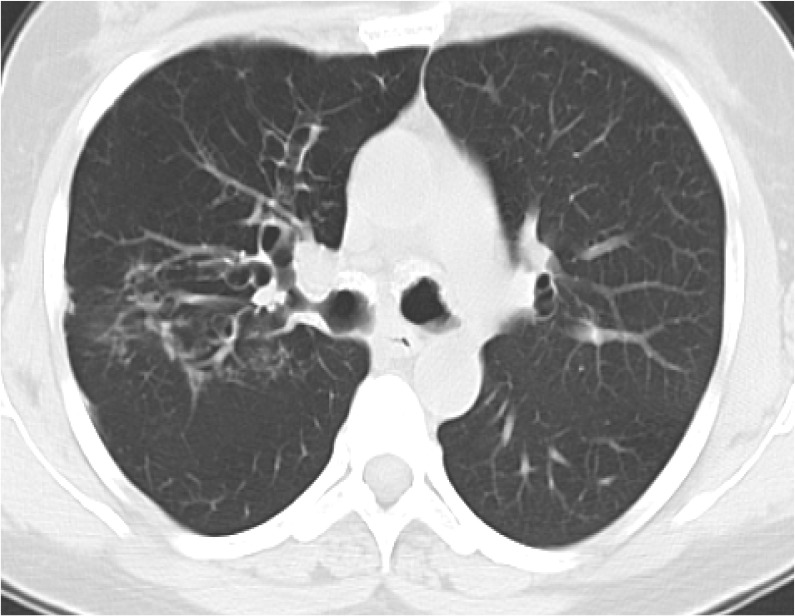
Chest tomography scan: thickening and calcification of the anterior bronchial walls main bronchi associated to diffuse and bilateral bronchiectasis

Recurrent lower respiratory tract infection was reported in our patient, which was treated by antibiotics. Thirteen years later, the patient was admitted for cough, dyspnea, and wheezing. Treatment with prednisone and inhaled bronchodilator was inefficient. Physical examination indicated diffuse sibilants, and the chest X-ray revealed the left lower lobe collapse. Moreover, the chest CT scan indicated the thickening and calcification of the anterior bronchial walls of the trachea and the main bronchi, associated with diffuse and bilateral bronchiectasis (Figure 2).

**Figure 3.**
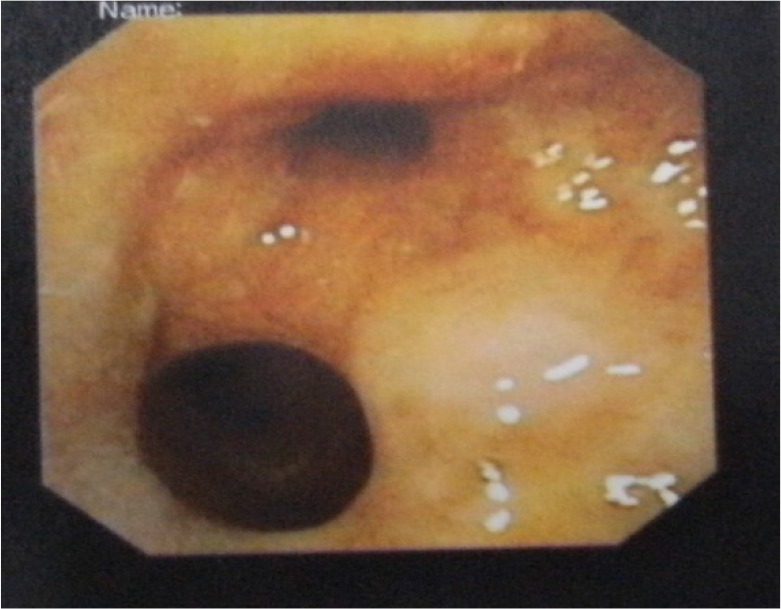
Bronchoscopy: nodular lesions distributed along the cartilaginous rings of lower portion of trachea and the main bronchi

**Figure 4.**
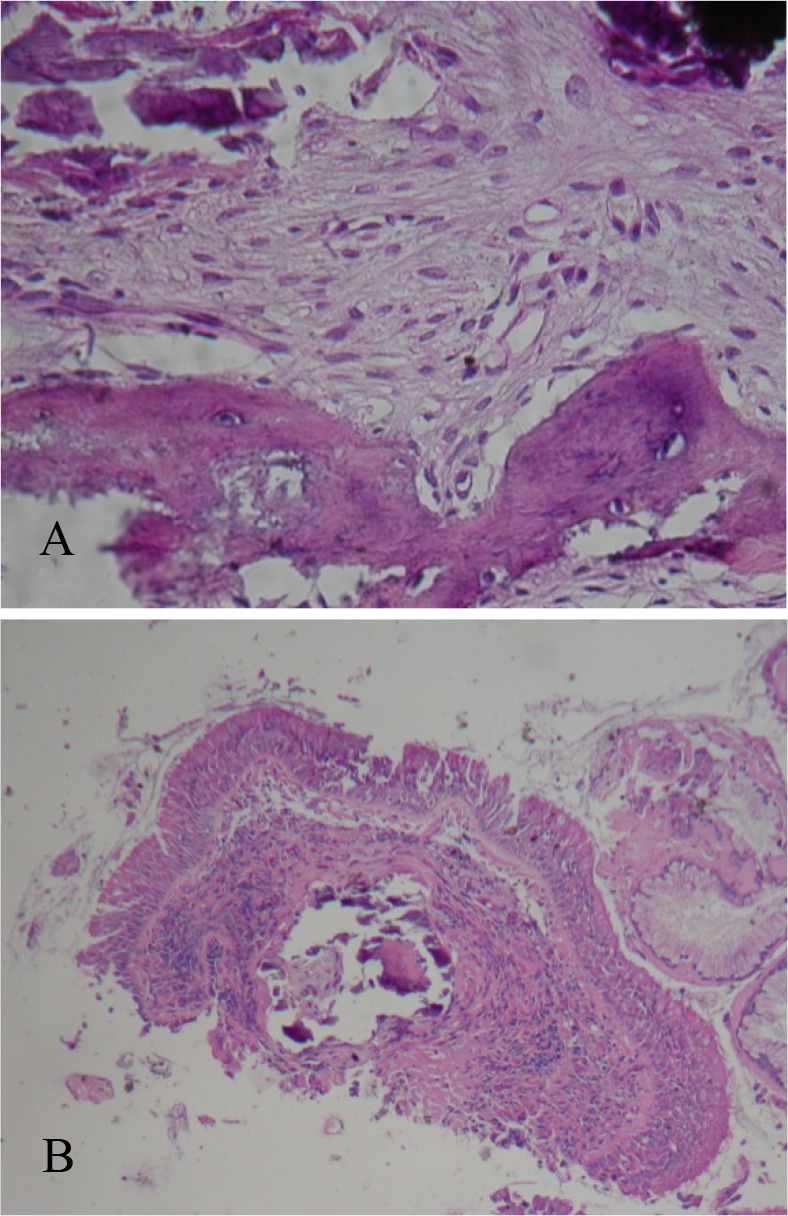
**(A, B)**. Endobronchial biopsy: Osseous formations in the bronchial submucosa with normal overlying respiratory epithelium

New fiberoptic bronchoscopy demonstrated nodular lesions distributed along the cartilaginous rings of the lower portion of the trachea and the main bronchi (Figure 3). Endobronchial biopsy revealed osseous nodules in the bronchial submucosa with a normal overlying respiratory epithelium, confirming TO (Figure 4 A,B).

## DISCUSSION

TO is a rare and benign disorder, characterized by cartilaginous and/or osseous submucosal deposits, projecting into the tracheobronchial lumen (1). This disorder is usually asymptomatic, and most cases are diagnosed incidentally via autopsy or bronchoscopy. TO is rarely diagnosed during difficult intubation (2, 3). This was first described by Wilks, a physician at Guy’s Hospital in 1857, who found that the larynx, trachea, and bronchi of a 38-year-old man (who died of tuberculosis) were covered with a number of bony plates in autopsy (2).

The exact incidence of TO remains unknown. To date, less than 400 cases of TO have been reported in the literature. This disorder is more common in adult males (>50 years), although it has been also reported in younger females and children (1,4,5). Although the etiopathogenesis of TO remains unknown, several theories have been proposed, including congenital deformity by Ribbert (1895), chronic infection by Wilks (1857), and chemical or mechanical irritation by Jackson and Jackson (1932). More recently, Sakula suggested connective tissue metaplasia, exostosis, and ecchondrosis from the cartilaginous trachea, followed by ossification and advanced ossified stage of primary localized amyloidosis of the lower respiratory tract (6), similar to our case. Also, a possible association with lung cancer was reported, although it seems to be coincidental (1).

Patients are usually asymptomatic, and symptoms are usually non-specific, including dry cough, dyspnea, recurrent respiratory infections, and occasionally hemoptysis (7). Chest radiography is normal in most cases, whereas irregularity and thickening of the trachea and calcification of the tracheobronchial tree or lobar collapse may be seen (1, 2). In our patient, chest X-ray showed the left lower lobe collapse. Generally, chest CT scan is an important imaging modality for diagnosis of TO, which can show calcified nodules, protruding into the tracheal lumen from the anterior and lateral walls of the trachea and sparing the posterior membranous portion (2, 8); this finding was also reported in our patient.

The endoscopic features of TO are typical and pathognomonic, consisting of sessile cartilaginous and/or bony nodules with normal overlying mucosa. These lesions are most frequently found in the distal two thirds of the trachea and major bronchi (1), similar to our patient. Diagnosis is based on a typical bronchoscopic appearance and does not generally require biopsy of the lesion. When available, histology can reveal bone formation within the submucosa with a normal overlying respiratory epithelium (7). Spirometry is often normal, but patients with a more extensive disease show a mainly obstructive pattern (3), similar to our case.

There is no specific treatment for TO. It is usually conservative consisting of antibiotics for recurrent respiratory infections. In case of failure, surgical treatment or interventional bronchoscopy can be discussed. Several surgical and bronchoscpic procedures have been used for TO, such as partial tracheal resection, partial laryngectomy, bronchoscopic removal of the lesion, stenting, and laser resection (1). The prognosis of TO is usually good and depends on the lesion extent and location. In advanced lesions, airway obstruction may induce obstructive pneumonitis and atelectasis (2).

## CONCLUSION

TO is a rare disorder with an unknown etiology and a commonly good prognosis. A relationship has been suggested between TO and primary localized amyloidosis of the lower respiratory tract. TO diagnosis is usually suspected in bronchoscopy and confirmed by histology. Sometimes, this disorder can lead to serious complications, requiring surgical or interventional endoscopic management.

## References

[1] Barthwal MS Chatterji RS Mehta A. Tracheobronchopathia osteochondroplastica. Indian J Chest Dis Allied Sci 2004;46(1):43–6. 14870868

[2] Tadjeddein A Khorgami Z Akhlaghi H. Tracheobronchopathia osteoplastica: cause of difficult tracheal intubation. Ann Thorac Surg 2006;81(4):1480–2. 16564296 10.1016/j.athoracsur.2005.04.013

[3] Thomas D Stonell C Hasan K. Tracheobronchopathia osteoplastica: incidental finding at tracheal intubation. Br J Anaesth 2001;87(3):515–7. 11517145 10.1093/bja/87.3.515

[4] Simsek PO Ozcelik U Demirkazik F Unal OF Orhan D Aslan AT Tracheobronchopathia osteochondroplastica in a 9-year-old girl. Pediatr Pulmonol 2006;41(1):95–7. 16284968 10.1002/ppul.20311

[5] Sant’Anna CC Pires-de-Mello P de Fátima Morgado M March MD. Tracheobronchopathia Osteochondroplastica in a 5-year-old Girl. Indian pediatrics 2012;49(12):985–6. 23315108 10.1007/s13312-012-0223-1

[6] Sakula A. Tracheobronchopathia osteoplastica: its relationship to primary tracheobronchial amyloidosis. Thorax 1968;23(1):105–10. 4966103 10.1136/thx.23.1.105 PMC471742

[7] Chroneou A Zias N Gonzalez AV Beamis J F Jr. Tracheobronchopathia osteochondroplastica. An underrecognized entity? Monaldi Arch Chest Dis 2008;69(2):65–9. 18837419 10.4081/monaldi.2008.398

[8] Restrepo S Pandit M Villamil MA Rojas IC Perez JM Gascue A. Tracheobronchopathia osteochondroplastica: helical CT findings in 4 cases. J Thorac Imaging 2004;19(2):112–6. 15071330 10.1097/00005382-200404000-00010

